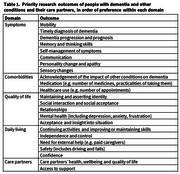# Research priorities for dementia and comorbid conditions: Co‐produced insights from people with dementia and care partners

**DOI:** 10.1002/alz70858_097253

**Published:** 2025-12-24

**Authors:** Lucy E Stirland, Rosalie Ashworth, Tom C Russ

**Affiliations:** ^1^ University of Edinburgh, Edinburgh, United Kingdom; ^2^ Global Brain Health Institute, University of California San Francisco, San Francisco, CA, USA; ^3^ NHS Lothian, Edinburgh, United Kingdom; ^4^ Neuroprogressive and Dementia Network, NHS Tayside, Dundee, United Kingdom; ^5^ Alzheimer Scotland Dementia Research Centre, University of Edinburgh, Edinburgh, United Kingdom

## Abstract

**Background:**

Although most people with dementia have several additional conditions, research often considers dementia, or its subtypes, in isolation. There is an ethical imperative to involve people with dementia in priority‐setting to ensure that resources are used on person‐centred topics.

Existing work has identified the research priorities of both people with dementia and – separately – people with multiple conditions. However, no studies to date have considered dementia in the context of multimorbidity from the perspective of affected people.

This project aims to set the research agenda by presenting new insights from people with dementia and comorbidities and care partners, co‐produced through focus groups.

**Method:**

We recruited two groups: people with dementia alongside multiple comorbidities, and care partners of people with dementia and multiple comorbidities. Inclusion criteria were living in Scotland, UK, and having video calling equipment. We held nine online focus group meetings: four for each group separately and one combined group. Meetings were facilitated by two experienced dementia researchers with expertise in co‐production.

The meetings comprised structured discussion of existing research outcome lists for dementia and multimorbidity. Each meeting was recorded and transcribed, then transcriptions qualitatively analysed between sessions using thematic analysis. This allowed for iterative development of a list of preferred outcomes, building on previous meetings.

During the final joint meeting, participants selected their top five research outcomes from the generated list.

**Result:**

We recruited five people with dementia (three of whom attended with a supporter) and four care partners. These group sizes were appropriate to the methodology and participant needs.

Iterative analysis of transcripts identified 23 priority outcomes that were categorised into symptoms, comorbidities, quality of life, daily living, and care partners (see Table 1). The top ranked outcomes were ‘continuing activities and improving or maintaining skills’, ‘mobility’, and ‘timely diagnosis’. Participants noted significant overlap between outcomes and emphasised that priorities vary between individuals, especially by stage of dementia.

**Conclusion:**

This Patient and Public Involvement study highlights the importance of comorbidities in dementia and the need for better recognition of this overlap in research and care. It has generated a list of person‐centred outcomes to inform future research.